# Metabolomics in the Prediction and Early Diagnosis of Type 2 Diabetes Mellitus: Advances and Applications

**DOI:** 10.7150/ijms.126351

**Published:** 2026-09-11

**Authors:** Xiaokai Wei, Min He, Haili Wang, Xin Xiang, Mengyuan Li, Mengmeng Li, Thanh Tuan Vu Le, Zhongxian Wang, Dingqi Zhang, Lei Cheng, Mengmeng Sun, Hongfeng Wang

**Affiliations:** 1 College of Acupuncture and Tuina, Changchun University of Chinese Medicine, Changchun, Jilin, China.; 2 Jilin Provincial International Cooperation Key Laboratory of Traditional Medicine for Prevention and Treatment of Metabolic Diseases, Changchun University of Chinese Medicine, Jingyue Economic Development District, Changchun, Jilin, China.; 3 Northeast Asia Institute of Traditional Chinese Medicine, Changchun University of Chinese Medicine, Changchun, Jilin, China.; 4 College of Traditional Chinese Medicine, Changchun University of Chinese Medicine, Changchun, Jilin, China.

**Keywords:** diabetes, metabolomics, prediction, early diagnosis, biomarkers, sample

## Abstract

Early detection and intervention can prevent the onset and progression of type 2 diabetes mellitus (T2DM); however, achieving early diagnosis and accurate prediction remains challenging. Metabolomics, a potent diagnostic tool in systems biology, makes it possible to systematically identify important biomarkers linked to type 2 diabetes, which provides vital proof for risk assessment and early diagnosis. This review summarizes research advances in metabolomics for T2DM prediction and early detection since 2010 by incorporating both the reported prospective and cross-sectional metabolic evidence, with a focus on putative biomarkers and their metabolic characteristics identified across diverse biological samples. Furthermore, it highlights the unique advantages of metabolomics in T2DM early warning, aiming to offer novel insights for future research on personalized prevention and early diagnostic strategies.

## 1. Introduction

Type 2 diabetes mellitus (T2DM), characterized by insulin resistance and a progressive loss of insulin secretion, is a chronic metabolic disease with a sharply increasing global prevalence. The International Diabetes Federation (IDF) estimates that 537 million adults had diabetes in 2021, a number projected to reach 783 million by 2045 1. Unmanaged hyperglycemia leads to severe micro- and macrovascular complications, profoundly impacting quality of life and imposing a substantial economic burden on healthcare systems 2. Therefore, the early and accurate identification of T2DM is crucial, and halting disease progression represents a key preventive strategy.

Prediabetes is the intermediary phase where blood glucose exceeds normal ranges but does not yet meet the diagnostic criteria for diabetes 3. This stage is potentially reversible, and precision or personalised nutritional interventions have been shown to improve cardiometabolic risk factors in this population 4. However, current diagnostic tools, including fasting plasma glucose, 2-hour postprandial glucose, and glycated hemoglobin (HbA1c), possess notable limitations. The sample source is restricted to human blood specimens; fasting blood glucose is temporally constrained, exhibits low sensitivity, and may result in overlooked diagnoses; postprandial blood glucose demonstrates inadequate stability and reproducibility; the oral glucose tolerance test necessitates multiple blood collections, is invasive, and is time-consuming, rendering it inappropriate for extensive population screening. In addition, HbA1c reflects average blood glucose levels over the preceding 2-3 months and may not capture acute glycemic fluctuations.

Human diabetes is a multifaceted disease shaped by environmental and genetic factors, often remaining asymptomatic for extended periods 5. Progression from prediabetes to T2DM can vary from months to over 10 years 6. Traditional risk factors like age, gender, and BMI offer limited predictive accuracy for the onset of T2DM 7. Consequently, there is an imperative necessity to identify precise biomarkers that can forecast the beginning of T2DM, enabling timely intervention.

Metabolomics, the study of endogenous small-molecule metabolites, offers a powerful solution. As an analytical method examining metabolic products and pathways, it provides detailed and dependable insights into T2DM onset and progression 8. Metabolomics has been effectively utilized for disease prediction and biomarker screening 9,10, as the differential expression of metabolites can elucidate molecular mechanisms at various stages of the disease 11. Over the past decade, metabolomics research in T2DM has advanced significantly. Initial studies often employed non-targeted techniques with small sample sizes to identify linked metabolites 12. This has progressed to large-scale, multi-cohort validations and mechanistic explorations 13. Recently, the focus has shifted to integrating multi-omics data and using advanced technologies like machine learning to develop robust prediction models from diverse biological samples such as blood, urine, and saliva 14,15.

Therefore, this review synthesizes metabolomics research since 2010 relating to T2DM prediction and early diagnosis across diverse clinical samples, by incorporating both the reported prospective and cross-sectional metabolic evidence. To achieve this, we will also summarize common methodological approaches and highlight critical research gaps that need to be addressed to advance the field. Ultimately, this review aims to provide a clear perspective on the current applications and future potential of metabolomics in the early management of T2DM.

## 2. Basic overview of metabolomics

Metabolites are endogenous small molecules with a molecular weight of less than 1000 Da that take part in metabolic processes within living organisms. They reflect comprehensive physiological and pathological changes in cells or tissues and constitute the ultimate outcomes of gene expression 16. Every metabolite found in a biological system during metabolic processes is included in the metabolome 17, and the chemical profile of a specific biological system serves as a reflection of its current phenotype. This profile is frequently utilized to analyze pathophysiological processes associated with disease progression and to investigate potential biomarkers for disease diagnosis or prognosis 18. Metabolomics constitutes a fundamental aspect of “omics” science, focusing on the analysis of chemical products or metabolites generated by cells and organisms. It investigates biological systems through the analysis of metabolite alterations resulting from stimulation or disruption of cells, tissues, or organisms. Metabolomics is recognized for its extensive capacity to predict phenotypes and has developed into a significant research tool in natural and life sciences 19, facilitating the understanding of biological system responses to internal and external stimuli 20. Metabolites, being the result of various interactions including those with the genome and proteome, enable metabolomics to accurately reflect an organism's phenotype with high sensitivity. This feature allows metabolomics to significantly contribute to the investigation of disease biomarkers (Figure 1). Metabolomics has offered a novel perspective on complex diseases, including diabetes, through ongoing research. Finding pertinent metabolites can help elucidate disease mechanisms and make it easier to find biomarkers for diagnosis and prognosis 21,22.

## 3. Methods in metabolomics research

The two primary categories of metabolomics research methodologies are targeted metabolomics and non-targeted metabolomics (Figure 2). Targeted and non-targeted metabolomics research techniques have significantly improved over the past decade.

Non-targeted metabolomics serves as the method of choice for hypothesis-free exploration. By achieving extensive metabolic coverage, this broad-spectrum strategy facilitates the bioinformatic identification of differential features and relevant pathways 23. It is particularly suited for discovering previously unidentified metabolites and novel biomarkers 24, thereby revealing new biological insights that might be missed by focused approaches 25.

Conversely, targeted metabolomics concentrates on the precise quantification of specifically known compounds 26. Frequently employed to validate non-targeted findings 27, this method integrates standard references to correct for matrix effects, enabling the accurate detection of low-abundance metabolites. It is essential for verifying specific disease correlations and elucidating pathophysiological mechanisms, especially in metabolic disorders, making it an indispensable tool for clinical diagnosis and biomarker investigation 28.

## 4. Technologies for metabolite detection

Analytical platforms such as chromatography, mass spectrometry (MS), nuclear magnetic resonance spectroscopy (NMR), and capillary electrophoresis (CE) are frequently employed in metabolomics research (Figure 3). When selecting the optimal detection technology, research goals must be carefully considered. Among these, NMR and MS—coupled with various separation techniques—dominate the field. Currently, the most prevalent methodologies are NMR, gas chromatography-mass spectrometry (GC-MS), and liquid chromatography-mass spectrometry (LC-MS).

NMR is widely utilized due to its non-destructive nature and capacity to concurrently identify numerous organic molecules in biological samples 29-31. This integrity-preserving characteristic makes it particularly suitable for stable isotope-labeled metabolic flux investigations and the analysis of valuable clinical samples, such as tissue biopsies. However, the technology faces challenges in effectively examining low-abundance metabolites due to limited resolution and sensitivity 32. Furthermore, the widespread application of NMR is restricted by its reliance on costly superconducting magnet equipment 33.

GC-MS is the preferred method for analyzing volatile, low-polarity, and thermally stable metabolites 34. While it offers high resolution for identifying specific compound classes like alcohols and organic acids, it is unsuitable for highly polar or thermally unstable substances in their native form 35. A significant operational challenge is the frequent requirement for chemical derivatization to increase sample volatility; this step complicates pretreatment, and compounds resistant to derivatization cannot be quantified.

LC-MS provides a broader analytical scope than GC-MS because the detection process is unaffected by sample volatility or thermal stability. Consequently, it is widely utilized for profiling complex biological fluids, including tissue extracts, blood, and urine 36-39. This platform eliminates the need for derivatization, thereby simplifying the workflow while delivering high sensitivity and throughput. Despite these advantages, the quantitative accuracy of LC-MS can be compromised by matrix effects, such as ion suppression or amplification 40.

For effective disease prediction or early diagnosis, the selection of biological samples and analytical platforms must be carefully tailored to the specific disease context and research objectives.

## 5. Metabolomics-based research for T2DM prediction and early diagnosis

### 5.1 Literature search and selection strategy

To identify relevant studies for background and comparison, a literature search was conducted across PubMed, Web of Science, and Embase for articles published between January 1, 2010, and March 31, 2026. The search utilized keywords such as "metabolomics," "type 2 diabetes," "prediabetes," "prediction," and "early diagnosis." The search was restricted to articles in English.

All retrieved records were imported into Zotero for duplicate removal. Two authors (X.W. and M.H.) independently screened the titles and abstracts of the remaining articles. Subsequently, the full texts of potentially eligible articles were assessed for final inclusion based on predefined criteria. Any disagreements during the screening process were resolved by a third author (H.W.).

To provide a comprehensive overview of metabolomics in 'early detection', our scope was designed to intentionally include two types of studies. The inclusion criteria were therefore formulated to capture both: (1) predictive studies, which investigate future (incident) T2DM risk in individuals healthy at baseline, and (2) diagnostic/cross-sectional studies, which identify biomarkers that distinguish individuals with prevalent prediabetes or early T2DM from healthy controls. We consider both study designs essential for a complete synthesis of the field.

Following this framework, the specific inclusion criteria were: (1) human studies (observational or interventional); (2) application of metabolomics for predicting future T2DM or diagnosing prediabetes/early T2DM; (3) reporting of specific metabolite biomarkers; and (4) original research articles.

Exclusion criteria were: (1) reviews, conference abstracts, or editorials; (2) animal or in vitro studies; (3) studies on type 1 or gestational diabetes; and (4) studies with a sample size < 20 or insufficient data for extraction.

The entire screening process is illustrated in the PRISMA flow diagram (Figure 4).

### 5.2 Characteristics of included studies

Following an extensive literature search, 66 studies were selected to representatively summarize the current progress in metabolomics applications for early T2DM diagnosis. These included both prospective and cross-sectional study designs. These studies represent a broad geographical and ethnic diversity, covering 19 nations across Europe, Asia, North America, and the Middle East, and including populations such as Han Chinese, Native Americans, African Americans, and multiethnic European cohorts. This global distribution underscores the growing interest in metabolomics for T2DM early diagnosis.

The sample sizes of the included studies varied widely, ranging from under one hundred to nearly one hundred thousand participants, reflecting a mix of small-scale exploratory studies and large-scale validation cohorts. The age range of subjects was also broad (14 to 90 years), with some studies focusing on specific age groups for targeted analysis. Notably, several recent studies specifically addressed gender differences in T2DM risk prediction, acknowledging the higher incidence rates observed in males 41,42 and contributing to personalized medicine approaches.

In terms of diagnostic criteria, the majority of studies follow recommendations from the American Diabetes Association (ADA) or the World Health Organization (WHO) (Table 1), which generally divide people into groups with prediabetes, healthy populations, and T2DM patients based on HbA1c, 2-hour postprandial blood glucose, and fasting blood glucose levels. Other studies have developed additional criteria specific to regional features or experimental needs.

The range of sample types includes metabolomics examination of biological samples, including blood, urine, saliva, feces and cerebrospinal fluid. Due to its simplicity of collection and good data stability, blood is the most commonly utilized sample among these; in contrast, cerebrospinal fluid is the least commonly used sample because of operational and ethical limitations in medicine. These diverse samples offer a multitude of information for clarifying the pathophysiology of T2DM as well as potentially acting as early disease diagnostic markers. Table 2 summarizes the general characteristics of the 66 included studies. By analyzing diverse biological samples, metabolomics not only yields novel insights for predicting T2DM but also provides critical evidence for designing and conducting subsequent clinical trials.

### 5.3 Heterogeneity in analytical platforms and data processing

Although these findings are encouraging, we observed significant methodological heterogeneity in data preprocessing, statistical analysis, and validation strategies, which exerts a crucial impact on the discovery and reproducibility of biomarkers.

In terms of data preprocessing, various methods were employed for missing value handling, including half of the minimum value 46, the k-nearest neighbor algorithm 47, and direct exclusion of metabolites with high missing rates. Normalization strategies were also inconsistent, with common approaches including total peak intensity normalization 48, internal standard correction, and batch correction based on quality control samples 49,50. For scaling and transformation, common approaches included log transformation 46, 51, Z-score standardization or Pareto scaling to improve data distribution 8, 12, 52. Technical differences in these preprocessing steps may introduce potential technical biases and affect the consistency of results across studies.

Regarding statistical analysis methods, a combined strategy of univariate and multivariate analyses was widely adopted 13, 53, 54: univariate methods, including t-test, Wilcoxon rank-sum test, and Mann-Whitney U test, were used for preliminary screening of differential metabolites; multivariate analyses extensively employed PCA, PLS-DA, and OPLS-DA for data dimensionality reduction and inter-group classification, with some studies further combining algorithms such as LASSO regression 55 and Random Forest to optimize feature selection 56. Notably, there were obvious discrepancies in multiple test correction strategies: when multiple-testing correction was reported, commonly used approaches included FDR or Bonferroni 13, 57, 58, while some studies did not clearly state the correction method. Such inconsistency in correction strategies may increase the risk of false-positive results.

In terms of validation, only a few studies conducted validation in independent external cohorts, and most relied on internal cross-validation or did not perform validation. This current situation highlights an important methodological bottleneck that metabolomic biomarker research needs to overcome before achieving generalizability and clinical translation. Future studies should promote the standardization of data preprocessing and statistical analysis workflows, and strengthen independent cohort validation to improve the reliability and clinical applicability of metabolic biomarkers.

### 5.4 Machine learning application status

In addition to the heterogeneity in analytical platforms and data processing mentioned earlier, machine learning has become a core analytical method in metabolomics-based T2DM prediction research.

Regarding algorithm application, logistic regression, random forest, and LASSO regression are the most commonly used. Logistic regression often serves as a stable and interpretable baseline model, with reported AUC values often falling in the range of 0.75-0.85 49, 55, 59. Random forest excels at handling nonlinear relationships in high-dimensional data, achieving AUC values above 0.85 in some studies through internal validation 56. LASSO regression, owing to its automatic feature selection capability, is widely used to screen key biomarkers from a large number of metabolites to construct concise predictive models. Furthermore, although partial least squares discriminant analysis and its variants are commonly employed for preliminary pattern recognition and biomarker discovery, they are mainly used for exploratory analysis and are less frequently used as final models subjected to rigorous external validation.

Model construction generally centers on metabolite features, with amino acids and lipid metabolites frequently reported as key predictors across studies 8, 49, 53. Some studies further integrated clinical features (such as age, BMI, and blood glucose indicators) 7, 49, 60. This integrative strategy typically enhances the biological plausibility and clinical utility of the models.

However, the rigor of model validation varies significantly, which directly impacts the assessment of their true efficacy. Among the 66 included studies, 15 (22.7%) have sample sizes of fewer than 100. Although such small-sample studies often report excellent performance in internal validation, most lack independent external validation or show significant performance degradation upon external testing, suggesting overfitting risks and limited generalizability. Overall, among the 66 studies included, approximately 74% rely solely on internal cross-validation, while only 17 studies have undergone rigorous independent external validation (Table 2). Study-level details, including sample size and study design for each of the 66 included studies, are provided in Supplementary Table S1. Across externally validated studies, findings were more consistently observed at the metabolic pathway level than at the individual metabolite level, with amino acid metabolism being the most frequently identified domain across different cohorts.

Furthermore, models incorporating a large number of metabolite predictors may be complex and costly to implement in routine clinical practice, potentially hindering their translation. There is also a paucity of head-to-head comparisons demonstrating whether metabolomics-based models provide incremental value over established clinical risk scores (e.g., based on age, BMI, and HbA1c). Therefore, future efforts should focus on constructing models within large-scale, multi-center cohorts, prioritize parsimonious models, and regard independent external validation as a necessary prerequisite for model evaluation and clinical translation, to enhance the robustness and generalizability of predictive tools.

## 6. Application of metabolomics in different samples for the prediction and early diagnosis of T2DM

Metabolomics research is inherently dependent on biological samples, whose characteristics, collection protocols, and extraction methods are key determinants 106. Different biological matrices present varying levels of accessibility and analytical challenges 18. Current T2DM research predominantly focuses on blood (plasma/serum), urine, saliva, cerebrospinal fluid, and feces (Figure 5). These matrices allow researchers to characterize metabolic perturbations induced by genetic and environmental factors, facilitating the identification of predictive biomarkers.

Building upon the analysis in the previous section, this discussion expands to a broader range of literature to comprehensively elucidate the applications of metabolomics across different biological samples. By delving into the physiological characteristics, detection advantages, and underlying mechanisms of each sample type, this section aims to provide a holistic perspective on their application potential for the early prediction of T2DM.

### 6.1 Application of blood metabolomics for predicting and early-diagnosing T2DM

In studies on metabolomics, blood is the most often utilized research sample. Along with being biomarkers, their associated metrics can be used to predict particular outcomes by acting as indications of physiological or pathological processes 107. Serum and plasma, the primary metabolite carriers of biological organisms, can provide a wealth of information regarding the physiological and pathological state of specific biological systems at specific times. Because serum and plasma are relatively easy to acquire and highly informative, their metabolites are commonly used for T2DM risk prediction and biomarker discovery 13, 67. For instance, the risk of T2DM is linked to amino acids, lipids, and carbohydrates 108. Therefore, serum and plasma represent ideal matrices for the early detection of a wide range of diseases.

Both plasma and serum are widely used in metabolomics, though they differ in preparation: plasma is collected with anticoagulants and retains clotting factors, whereas serum is obtained after clotting and centrifugation. Some evidence suggests that plasma may better preserve certain labile metabolites, while the clotting process in serum preparation may introduce additional variability 109. Among the studies reviewed here, both matrices yielded largely comparable findings. Core biomarkers, including the branched-chain amino acids (BCAAs), particularly leucine and valine, the aromatic amino acid phenylalanine, glycine, serine, and α-hydroxybutyric acid (also known as 2-hydroxybutyric acid), showed largely consistent directional changes across plasma- and serum-based studies. However, a small number of metabolites, such as lysine and arginine, showed inconsistent trends between the two matrices in our synthesis 54, 70, 88, 89, which may reflect differences in sample preparation, study populations, or analytical platforms. Notably, no studies in our review performed a direct within-cohort comparison of serum versus plasma. Even the few studies that utilized both matrices did so for cross-cohort purposes, such as combining results or validating models, rather than for a direct comparison 13, 102.

Regarding amino acid metabolism, a highly robust finding is the alteration of several key amino acids. A study by Jabbar Al-Rikabi et al. 69 identified significant differences in glycine, serine, and proline between prediabetes and normal groups. In another representative study, Wenrui Ji et al. 11 identified L-leucine and L-arginine as biomarkers of prediabetes progression. The elevation of BCAAs like leucine is widely thought to interfere with insulin signaling 110, while changes in glycine and serine may reflect disruptions in oxidative stress defense and insulin sensitivity 111, 112. This consistent pattern of amino acid dysregulation across multiple cohorts highlights its central role in early T2DM pathogenesis.

In the lipid domain, the accumulation of specific acylcarnitines is frequently reported, and this suggests incomplete mitochondrial fatty acid oxidation 8, 54, 85. Furthermore, other novel biomarkers linked to energy metabolism and related pathways have been identified. For instance, α-hydroxybutyrate (α-HB) has emerged as a powerful early marker of insulin resistance 86, 113. In the same line of research, peptide metabolites such as hydroxyproline-kynurenine ([Hyp3]-BK) were also identified as potential biomarkers. As a modified bradykinin, [Hyp3]-BK is involved in regulating vascular function, and its alteration may be linked not only to T2DM risk but also to future microvascular complications 114. Other markers like mannose have also been noted, potentially indicating disturbances in protein glycosylation pathways 86, 115.

In summary, blood metabolomics provides a powerful and consistent set of biomarkers for T2DM prediction. While the amino acid signature—particularly BCAAs—is remarkably robust, some variability exists in reported lipid biomarkers. This discrepancy can often be attributed to methodological differences. For instance, despite notable differences in the overall biomarker profiles identified by prospective (identifying future risk) versus cross-sectional (reflecting the current disease state) studies, a consistent directional trend is nevertheless often reported for certain key metabolites, such as elevated BCAAs and reduced glycine 48, 60. Furthermore, the choice of analytical platform plays a crucial role; LC-MS-based approaches excel at identifying a wide array of individual species, whereas NMR-based analyses focus on broader lipoprotein profiles, leading to complementary sets of biomarkers. Despite these variations, integrating blood-based metabolic markers with established clinical risk factors holds great promise for improving early and accurate risk assessment 8.

### 6.2 Application of urine metabolomics for predicting and early-diagnosing T2DM

Urine can be collected non-invasively in relatively large volumes, making it well-suited for metabolomics studies that require substantial sample sizes 116. Urine metabolomics has been applied to a range of conditions, including diabetes 117, cancer 118, autism 119, and liver disease 120. Although urine metabolomics has been less extensively studied than blood in the context of T2DM biomarker development, preliminary findings suggest it may provide complementary metabolic information for risk assessment and early diagnosis 12.

Reflecting systemic metabolic stress, urinary levels of acylcarnitines and BCAA catabolites have been identified as potential biomarkers. For instance, Salihovic et al. 121 proposed urine 3-hydroxyundecyl carnitine as a biomarker, while another study found the BCAA metabolite 3-methyl-2-oxopentanoate (3-MMPO) to be a strong predictor for impaired fasting glucose 82. These findings mirror the mitochondrial dysfunction observed in blood studies.

Urine metabolomics also effectively reflects contributions from the gut microbiome. A study by Xinjie Zhao et al. 12, for instance, identified that urine concentrations of several metabolites linked to gut microbiota activity, such as methylxanthine, methyluric acid, and hippuric acid, were lower in people with impaired glucose tolerance. This highlights how urine can serve as a valuable, non-invasive matrix for capturing metabolic signals related to gut dysbiosis.

Furthermore, findings from urine metabolomics have underscored the importance of gender-specific analysis. A notable study by Nele Friedrich et al. 122, for example, found distinct sets of predictive urinary metabolites in men and women. In women, metabolites like acetic acid and carnitine were predictive, while in men, valine and 4-hydroxyphenylacetic acid were key biomarkers. This work underscores the need for gender-stratified analysis in future biomarker research.

In summary, given the limited number of urine metabolomics studies identified in this review, current evidence is preliminary. Larger, particularly longitudinal, cohorts are needed to advance biomarker discovery and validation. A major challenge in this field is the high variability introduced by diet, medication, and hydration status, which, combined with inconsistent collection and normalization protocols, contributes to discrepancies across studies. Therefore, future efforts could prioritize standardized operating procedures and rigorous validation in large, well-controlled longitudinal studies to help establish the clinical utility of urinary biomarkers.

### 6.3 Application of salivary metabolomics for predicting and early-diagnosing T2DM

Saliva is a colorless, thin, and slightly viscous fluid generated in the oral cavity. It is slightly acidic and is mostly made by three pairs of major salivary glands: the parotid, sublingual, and submandibular glands, as well as a few smaller salivary glands 123. Like blood testing, saliva testing can reflect systemic health and disease status 124. Salivary metabolomics examines dynamic changes in small-molecule metabolites to identify biomarkers for oral diseases 125,126, systemic diseases 127-129, and individual health variations 130. Owing to its straightforward, rapid, cost-effective, and non-invasive collection 131, saliva enables repeated sampling with minimal adverse effects and is highly suitable for large-scale population screening 132. Although salivary metabolomics has been applied to various conditions—including periodontitis 133, diabetes 134, Parkinson's disease 135, cancer 136, and Sjögren's syndrome 137—studies specifically focused on T2DM prediction remain relatively scarce.

Saliva can mirror key metabolic changes of T2DM. A cross-sectional study by Virginia M. Barnes et al. 97 indicated that saliva from diabetic individuals showed elevated glucose, increased α-HB, and reduced levels of 1,5-Anhydroglucitol (1,5-AG) compared to non-diabetic controls. A similar pattern was observed by Noha A. Yousri et al. 98, who also noted changes in amino acids like alanine and glutamate.

The reduction of salivary 1,5-AG, a well-established marker of short-term glycemic control, shows promise for non-invasive glucose monitoring 138. However, research in salivary metabolomics for T2DM prediction remains nascent. The current scarcity of large-scale longitudinal studies precludes the identification of a validated, saliva biomarker panel. Therefore, despite the ease of collection which makes saliva ideal for screening, extensive validation in large, diverse cohorts is urgently needed to confirm the clinical reliability and utility of these findings 139.

### 6.4 Application of cerebrospinal fluid (CSF) metabolomics for predicting and early-diagnosing T2DM

Cerebrospinal fluid (CSF) is closely associated with the central nervous system (CNS) and therefore provides a useful matrix for metabolomic investigation. The mean CSF volume is approximately 150 mL, which is renewed approximately four times every 24 hours 140. Because CSF enables metabolic exchanges between blood and the brain 141 and is directly connected to the brain and spinal cord, CSF metabolomics may more accurately reflect physiological alterations within the CNS than other sample types. Consequently, it is considered important for understanding disease mechanisms and drug discovery 142, and is more relevant to neurological disorders than peripheral samples 143. Notably, only one study on CSF metabolomics in T2DM was identified in this review.

This single study revealed a distinct metabolic signature in T2DM patients compared to controls, characterized by higher levels of mannose, lactic acid, pyruvic acid, and several amino acids, while histidine was reduced 101. These findings, some of which appeared more pronounced in CSF than in plasma 101, point to heightened glycolysis and amino acid dysregulation within the CNS, offering a unique window into the metabolic state of the brain during T2DM development.

While these preliminary findings are promising, the practical application of CSF metabolomics is restricted by the invasive nature of lumbar puncture, which limits studies to small sample sizes 144. This scarcity of data makes it difficult to draw robust conclusions or compare findings across studies. Therefore, future progress in this area will likely depend on multi-center collaborations to increase sample sizes and validate these promising but preliminary findings.

### 6.5 Application of fecal metabolomics for predicting and early-diagnosing T2DM

Fecal metabolites correlate with age, gender, and body fat 145, providing valuable insights for disease diagnosis and treatment monitoring 146. In T2DM, shifts in intestinal microbiota abundance alter metabolites—including branched-chain amino acids, short-chain fatty acids, and bile acids 147—which may drive disease progression. Thus, fecal metabolomics is a promising resource for early intervention, though only two studies were identified in this review.

One key study by Rebiya Nuli et al. 99 revealed significant shifts in fecal lipid composition, particularly fatty acyl and glycerophospholipid compounds, across normal, impaired glucose regulation, and T2DM groups. This indicates that abnormal host lipid metabolism is reflected in fecal contents. Furthermore, integrating fecal metabolomics with gut microbiota analysis is a valuable strategy for understanding T2DM pathogenesis 100, as it clarifies how microbial shifts drive metabolic changes that contribute to diabetes. This combined approach offers critical information for both diagnostics and targeted interventions.

In summary, fecal metabolomics provides a direct window into the gut microbiota-host axis. Moreover, evidence from fecal metabolomics remains limited. The fecal metabolomics study analyzed in this review employed a cross-sectional design 99, which restricts causal inference. Diet and sample handling may also introduce variability. Therefore, future progress will rely on larger, well-designed longitudinal studies with standardized protocols to improve reproducibility and clinical relevance.

## 7. Discussion and Outlook

Our synthesis of 66 human studies shows that the metabolic signature of each biological sample has its own distinct features, and these features largely determine how useful that sample can be for early prediction and diagnosis of T2DM (Figure 6, Table 3). In Table 3, serum and plasma are presented separately for detailed reporting, whereas in Figure 6 they were combined into the single category “blood” to provide an integrated comparison across sample types. Blood is by far the most extensively studied sample type. Its core metabolic markers have been repeatedly reported across populations, making blood-based metabolomics a relatively mature area for potential clinical translation. By contrast, only a very limited number of studies examined urine and fecal metabolomics. Preliminary evidence suggests that urinary metabolites may reflect renal handling of glucose and related metabolic perturbations, whereas fecal metabolites may offer insight into gut microbiota-host interactions in T2DM development. Although only a small number of salivary metabolomics studies have been identified, these studies generally suffer from small sample sizes and limited analytical depth. Despite this, preliminary evidence indicates partial overlap of certain metabolites between saliva and blood, consistent with Figure 6, where salivary metabolites were shared with blood rather than being saliva-unique, suggesting potential for non-invasive screening. Cerebrospinal fluid metabolomics may provide additional information on central nervous system metabolism in T2DM; however, because only one CSF study was identified, its relevance for early prediction or diagnosis remains highly preliminary. In summary, different biological matrices may offer complementary metabolic perspectives, but for non-blood samples this possibility remains insufficiently validated because of the limited number of available studies.

A summary of the 66 studies in Table 3 shows that valine and phenylalanine were among the most frequently reported upregulated metabolites, whereas glycine was frequently reported as downregulated, particularly in blood-based studies. However, the direction of glycine change was not uniform across all sample types, as an opposite trend was reported in CSF. Branched-chain and aromatic amino acids showed relatively consistent directional trends across studies, although matrix-specific variability was observed for some metabolites, suggesting that they represent some of the most consistently reported candidate biomarkers for early-stage T2DM.

It should be noted that predictive and diagnostic studies (prospective and cross-sectional studies) reflect biologically different disease states. Prospective studies relate to future risk prediction, whereas cross-sectional studies relate to the diagnosis of prevalent prediabetes or early T2DM. However, taken together, they offer complementary insights into metabolic changes relevant to early detection of T2DM. In this review, the study design of each included article is explicitly indicated in Supplementary Table S1, which provides a transparent overview of key study-level information.

To further analyze the expression characteristics of metabolic biomarkers across different samples, we screened core metabolites that appeared in two or more sample types from 66 included studies and constructed an expression characteristic heatmap (Figure 7). The strength of evidence for each metabolite varies across the literature. For example, phenylalanine 54, 57, 64, 67, 88-90, 98, 101, alanine 54, 63, 64, 68, 90, 98, 101, 103, and valine 48, 54, 57, 67, 69, 90, 101, 103 were reported in 9, 8, and 8 independent studies, respectively, indicating relatively consistent findings across studies. These key metabolites were reported across both study designs. This may suggest that the metabolic dysregulation they reflect, particularly in amino acid metabolism, is not restricted to a specific disease stage. Instead, it appears to be a continuum from early insulin resistance to established T2DM. Therefore, these markers may be valuable both for predicting future risk and for diagnosing metabolic disruption in its early clinical phases. With this context in mind, the heatmap suggests several patterns, although the level of supporting evidence differs markedly among metabolites and sample types. In the amino acid pathway, leucine, valine, and tyrosine were upregulated in both blood and cerebrospinal fluid, whereas glycine showed opposite directions between blood and CSF. In lipid metabolism, lysophosphatidylcholine was downregulated in blood and urine but upregulated in feces. Among carbohydrate-related metabolites, glucose and 1,5-AG showed similar directional changes in blood and saliva, while mannose exhibited opposite trends. Uric acid was increased in blood but decreased in urine. However, it should be noted that findings for urine, saliva, fecal, and CSF matrices are based on a very limited number of studies; therefore, these cross-matrix patterns should be interpreted with caution and require further validation in larger cohorts.

The distinct metabolic characteristics offer significant evidence for the multidimensional prediction of T2DM. Metabolomics testing results are influenced by factors including individual differences 61,56, sample type 82,101, collection time 55, and gender 122, demonstrating distinct personalized characteristics. The observed differences may arise from multiple factors, such as sample collection procedures, methods, timing, fasting status, processing and storage conditions, and testing techniques. In metabolomics research, it is essential to strictly control experimental variables and standardize operational procedures to ensure the reliability of results.

Currently, metabolomics has advanced considerably in the early prediction and diagnosis of T2DM; however, several limitations persist. Firstly, current research predominantly employs a singular sample type, with a heavy emphasis on blood analysis, while other biological samples that permit non-invasive collection receive inadequate attention. This limitation may impede the thorough investigation of metabolic biomarkers associated with diabetes. Secondly, metabolomics research is characterized by a lack of standardization in sample collection and testing protocols, resulting in inconsistencies in the identification of essential biomarkers. Variations in the process increase the incomparability between studies and interfere with the determination of diabetes biomarkers. Finally, due to medical ethical constraints, the routine acquisition of certain invasive samples in human studies is challenging. Consequently, metabolomics data from cerebrospinal fluid, tissue biopsies, and other sources predominantly derive from animal experimental models 148. Sample acquisition limitations present challenges to the discovery and validation of disease-specific metabolic markers in humans, potentially impacting the reliability of research conclusions. In addition, as a narrative review, the included studies varied substantially in design, sample type, analytical platform, and study objectives; therefore, a formal standardized risk-of-bias assessment using a single tool was not performed.

To advance metabolomics research for the prediction and early diagnosis of T2DM, future studies should focus on addressing existing challenges in the following areas:

(a) Explore diverse biological samples. Most current human studies predominantly use blood (serum/plasma), whereas only a very small number investigate urine, saliva, feces, or cerebrospinal fluid. At present, available evidence is insufficient to determine whether combining multiple sample types improves T2DM prediction compared with single-matrix approaches. Given that different biofluids may reflect distinct metabolic processes, exploring multi-sample strategies therefore emerges as a particularly promising research direction. Future studies should investigate, in adequately powered cohorts, whether biomarker combinations across different matrices can improve early diabetes prediction beyond blood-based markers alone.

(b) Promote standardized operational procedures. A key finding from our review is that methodological heterogeneity may partly account for inconsistencies in reported biomarkers. Standardization is important because differences in sample matrix, collection time, fasting status, storage conditions, analytical platform, and data processing can directly affect the comparability of metabolomic findings across studies, thereby limiting biomarker reproducibility. For example, in serum, phenylalanine was reported as upregulated when analyzed by GC-MS or LC-MS 90,57, but downregulated when NMR was used 88; similarly, in plasma, alanine was found to be upregulated by NMR 101, yet downregulated by FIA-MS/MS 68. Although these discordant findings may also reflect differences in population characteristics, fasting status, or study design, they suggest that analytical platform and pre-analytical handling can materially influence the reported associations. However, because these discordant findings were derived from studies conducted in different countries and populations, the current literature does not allow us to determine definitively whether the observed inconsistencies are primarily attributable to analytical methods rather than biological differences. In addition, many included studies did not provide sufficient detail regarding collection time, storage temperature, storage duration, freeze-thaw conditions, metabolite identification standards, calibration procedures, or quality-control workflows, making it difficult to determine whether between-study differences reflect true biological variation or technical variation. Future dedicated methodological studies or reviews are needed to systematically address this gap and to establish consensus guidelines for pre-analytical and analytical standardization in T2DM metabolomics research.

(c) Strengthen animal model research. This review focused exclusively on human studies, and animal model research was not systematically assessed. However, several limitations identified in the reviewed literature highlight the need for animal studies. While numerous candidate biomarkers have been associated with T2DM risk in observational human cohorts, the causal metabolic pathways underlying these associations remain largely unclear. Animal models can circumvent the ethical and technical constraints associated with acquiring invasive human tissues and facilitate mechanistic investigations under controlled experimental conditions. Future research should clarify which specific biomarker-pathway relationships identified in human metabolomics studies require validation through animal experiments, particularly for metabolites whose mechanistic roles in insulin resistance and beta-cell dysfunction remain speculative.

(d) Advance the integration of personalized medicine and TCM. Among the reviewed studies, metabolomic analyses were predominantly conducted at the population level, with only a few studies attempting to perform personalized analyses. This represents a significant limitation, as metabolite profiles are known to vary considerably across these subgroups. Currently, the clinical application of metabolomics-based personalized medicine for T2DM remains in its infancy, and no study in this review demonstrated a complete pathway from biomarker discovery to individualized clinical decision-making. One emerging approach in this direction is the integration of metabolomics with Traditional Chinese Medicine (TCM) syndrome differentiation. Studies by Wei et al. 52 and He et al. 149 have attempted to identify metabolic subtypes corresponding to specific TCM syndromes in prediabetic populations, suggesting that metabolomics may provide an objective molecular basis for TCM classification. However, these efforts remain preliminary, with small sample sizes and limited external validation. Future research should further explore this synergy in larger, more diverse populations to enhance the precision of early diabetes prediction and advance personalized prevention strategies.

(e) Integrate multi-omics data and machine learning. Currently, the research frontier is expanding from single-omics to the integrated analysis of multi-omics data. For example, a recent study successfully constructed a model to predict diabetes progression by integrating metabolomic, lipidomic, and proteomic data and applying multiple machine learning algorithms, including LASSO, ridge regression, and random forests 150. This study confirmed that even though models built using classical clinical indicators such as age, BMI, and HbA1c already demonstrate good predictive performance, the discriminative ability of the models can be further enhanced by incorporating multi-omics molecular information, thereby verifying the independent incremental predictive value of molecular markers relative to traditional risk factors. To strengthen the molecular foundation for early intervention, future research should focus on promoting deeper integration of multi-omics data and the continuous optimization of intelligent predictive models 151. In this process, rigorous external validation in independent cohorts is essential for improving the reliability of risk assessment and advancing the clinical translation of precision interventions.

(f) Accelerate clinical translation of metabolomic findings. A critical gap identified in this review is the distance between biomarker discovery and clinical application. Among the human studies reviewed here, a small number conducted external validation in independent cohorts, but none progressed to the development of clinically deployable diagnostic tools or implementation in clinical practice. Clinical translation requires additional steps including large-scale multi-center validation, regulatory approval, and industry collaboration, processes beyond the scope of typical metabolomics research. While commercial assays such as the Quantose™ IR test 152 have demonstrated feasibility, widely accessible tools like point-of-care devices remain scarce 153. Future efforts should prioritize multi-center validation of consistently identified biomarkers and foster collaboration among researchers, clinicians, and industry partners to bridge the gap between discovery and clinical application.

## 8. Conclusion

In the study of T2DM, metabolomics has emerged as a pivotal tool for early diagnosis and disease prediction. To identify novel biomarkers, elucidate underlying mechanisms of pathogenesis, and clarify the causal relationships of metabolic abnormalities, future research must prioritize conducting additional multicenter, large-scale cohort studies. These studies should investigate metabolic profiles across diverse populations and distinct disease stages. Ultimately, this will be instrumental in translating metabolomic discoveries from basic research into clinical practice, enabling early warning for T2DM.

## Supplementary Material

Supplementary table.

## Figures and Tables

**Figure 1:**
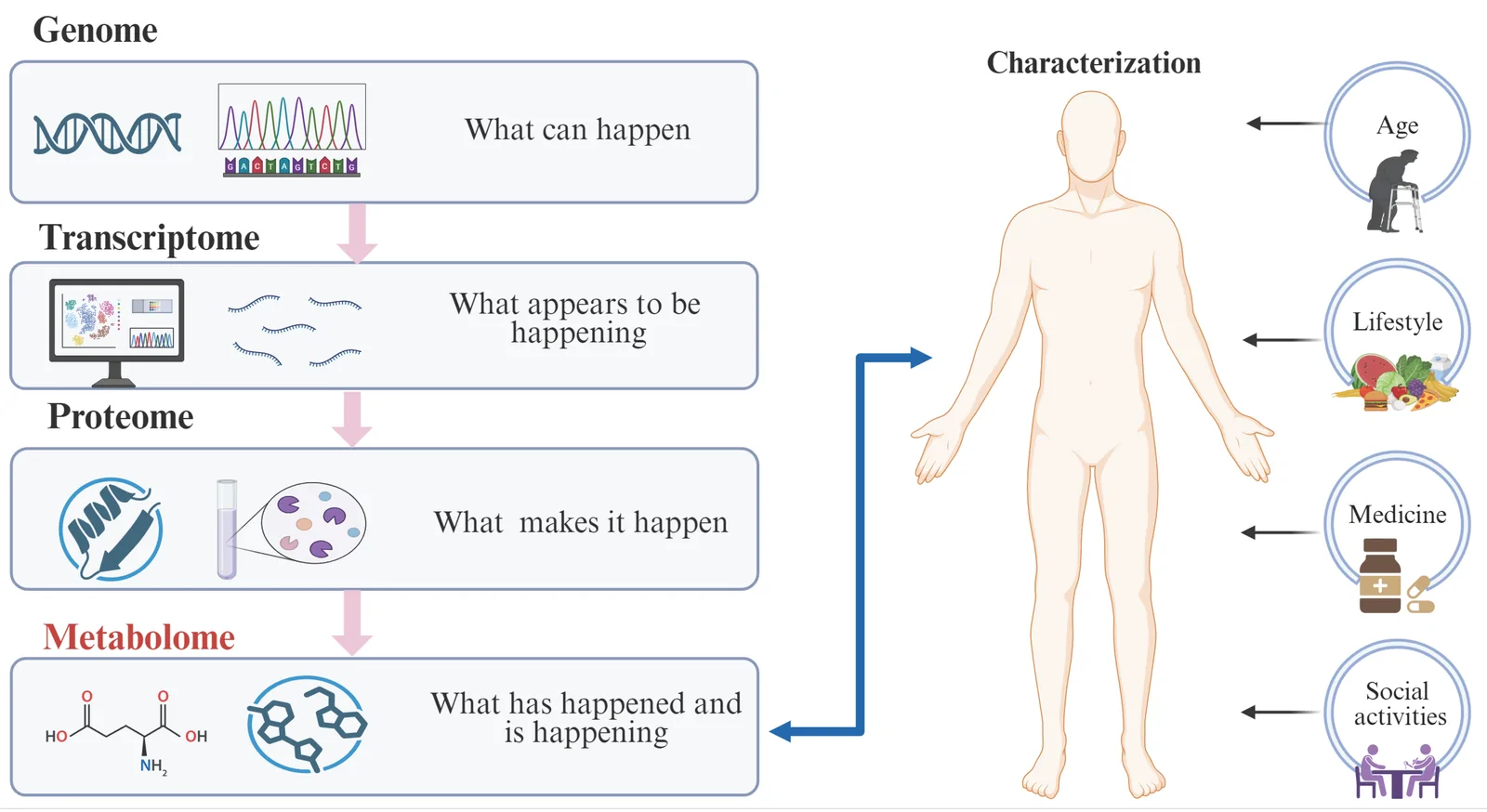
** Metabolomics and other omics in relation to each other.** Overview of the relationship between metabolomics and other omics layers, showing that the metabolome reflects physiological and pathological states and is influenced by multiple factors. Created with BioRender.com (Agreement number: HY29M9Q500).

**Figure 2:**
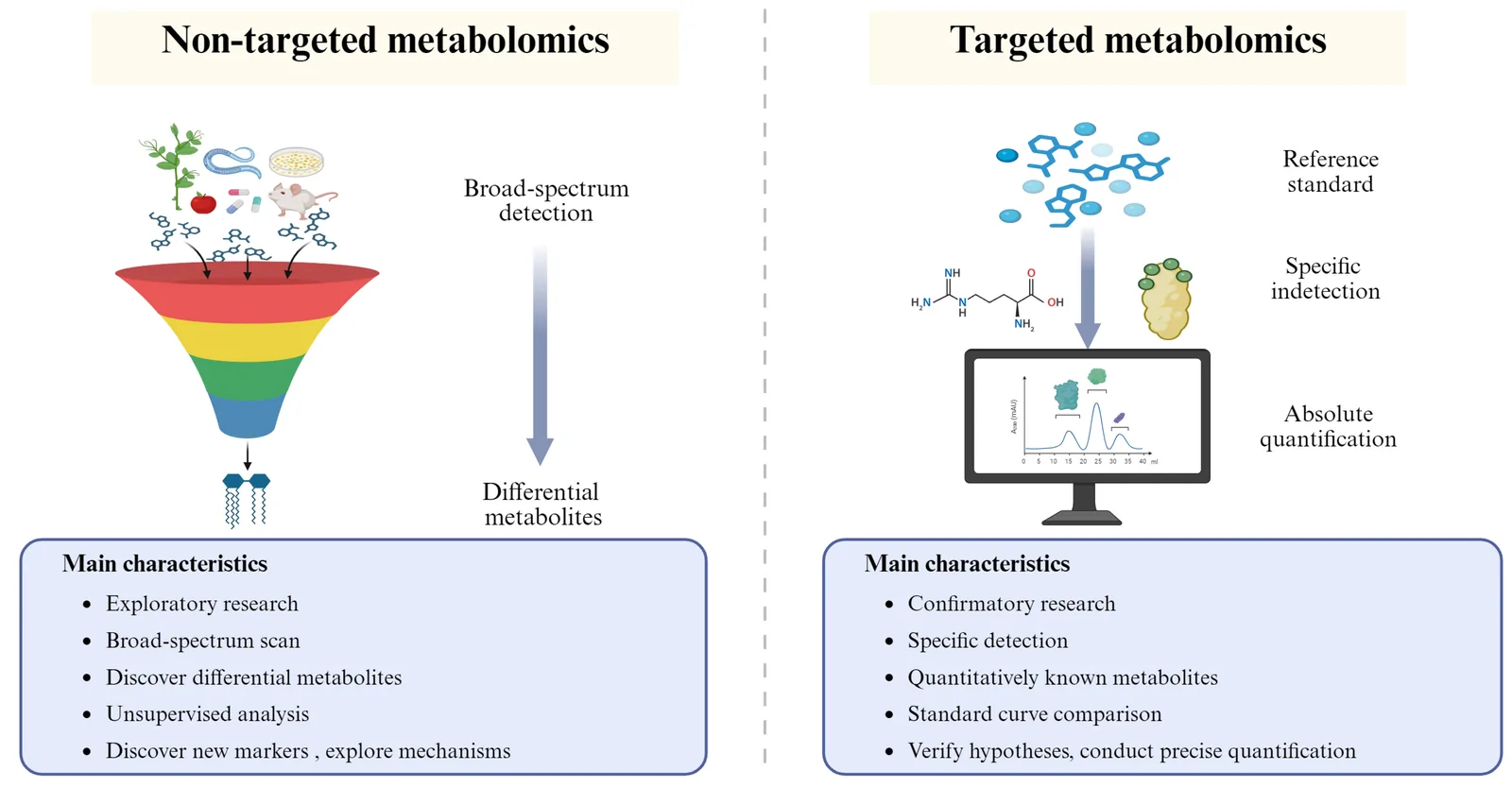
** Non-targeted metabolomics and targeted metabolomics.** Comparison of the methods and main characteristics of non-targeted and targeted metabolomics. Created with BioRender.com (Agreement number: AM29M9Q3XZ).

**Figure 3:**
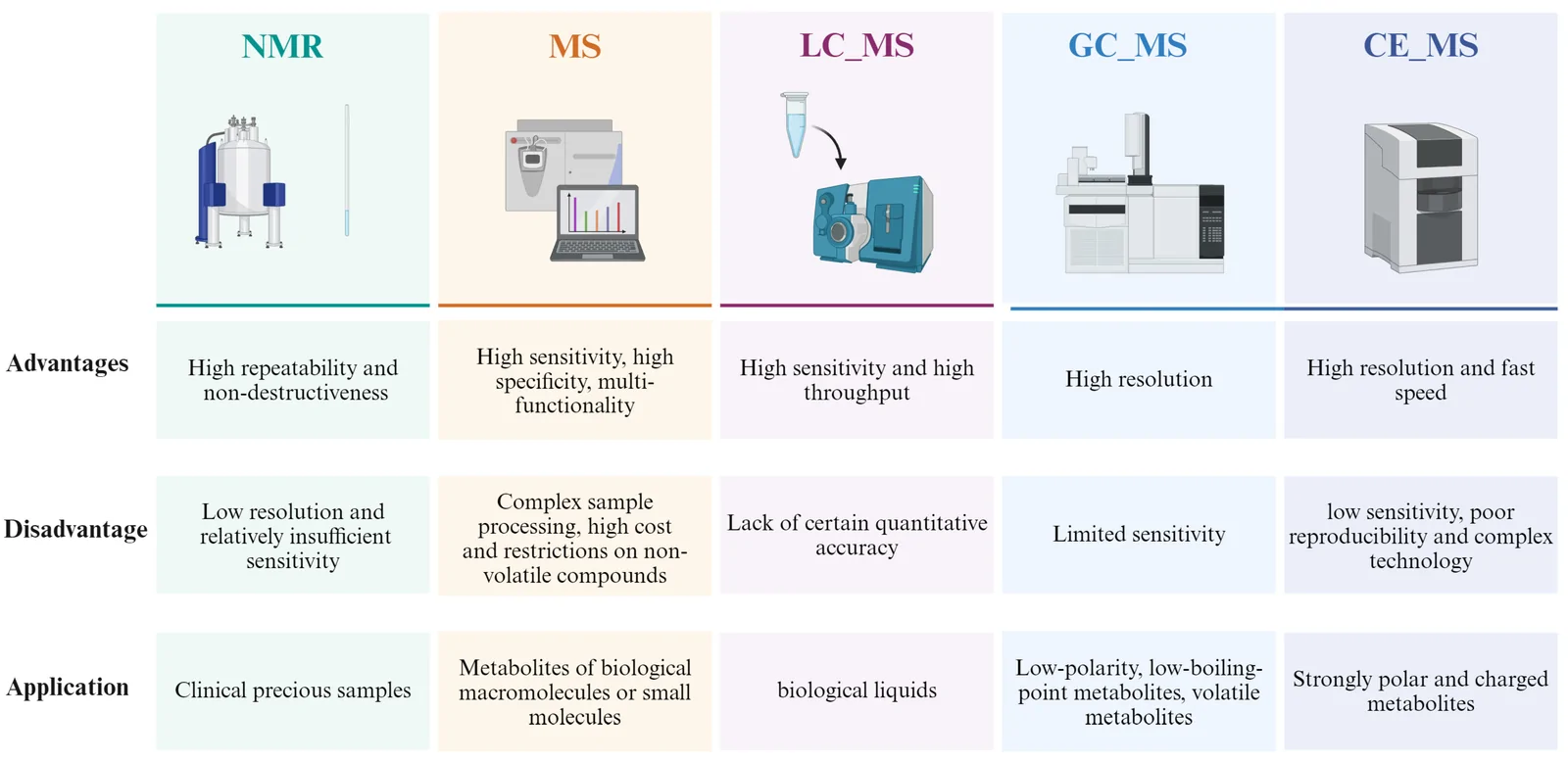
** Frequently employed metabolomics methods.** Common analytical platforms used in metabolomics, together with their respective characteristics and areas of application. Created with BioRender.com (Agreement number: UF29M9PZWE).

**Figure 4:**
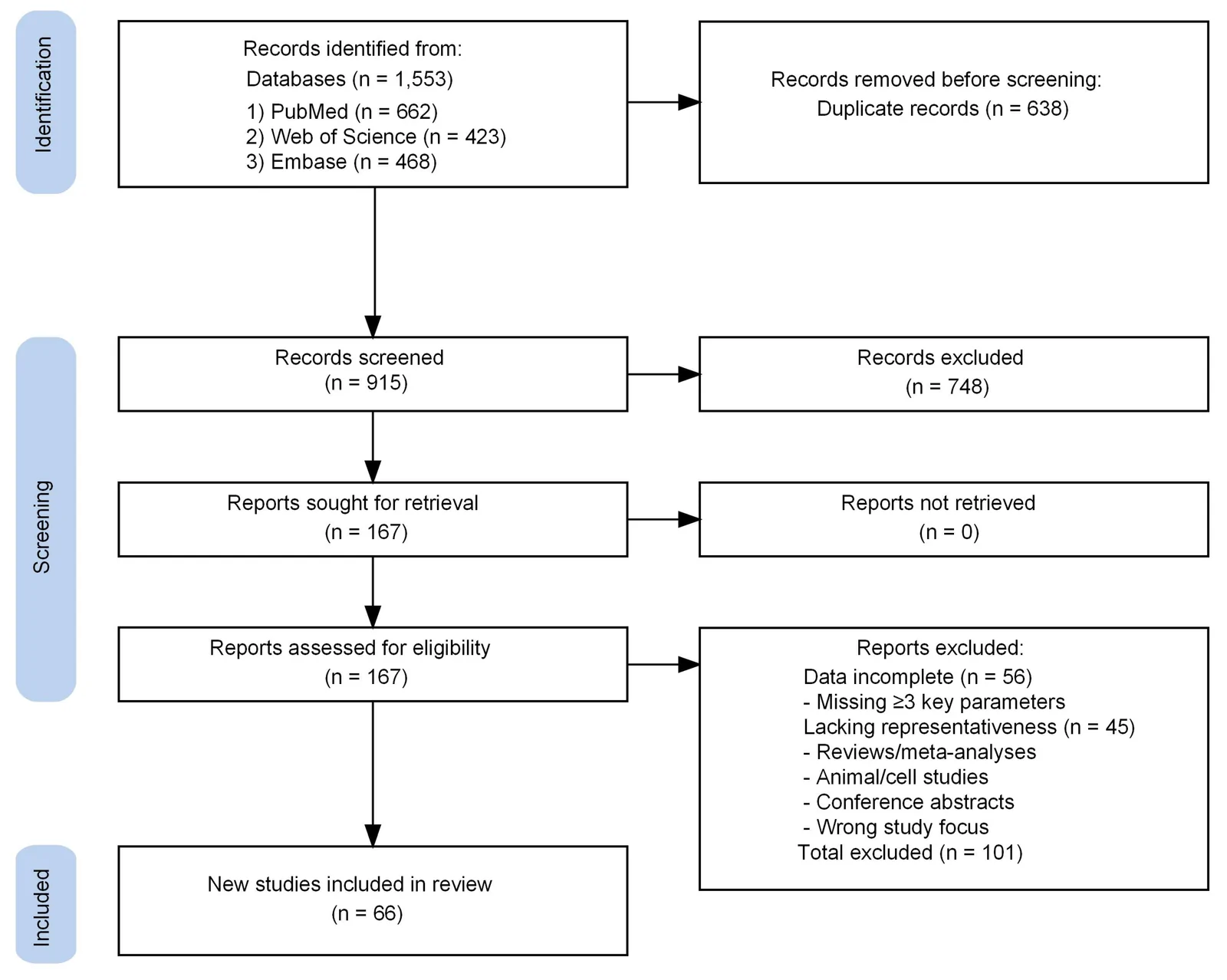
** Flowchart of the literature selection process.** PRISMA flowchart illustrating the process of literature retrieval, screening, eligibility assessment, and final inclusion of studies in this review.

**Figure 5:**
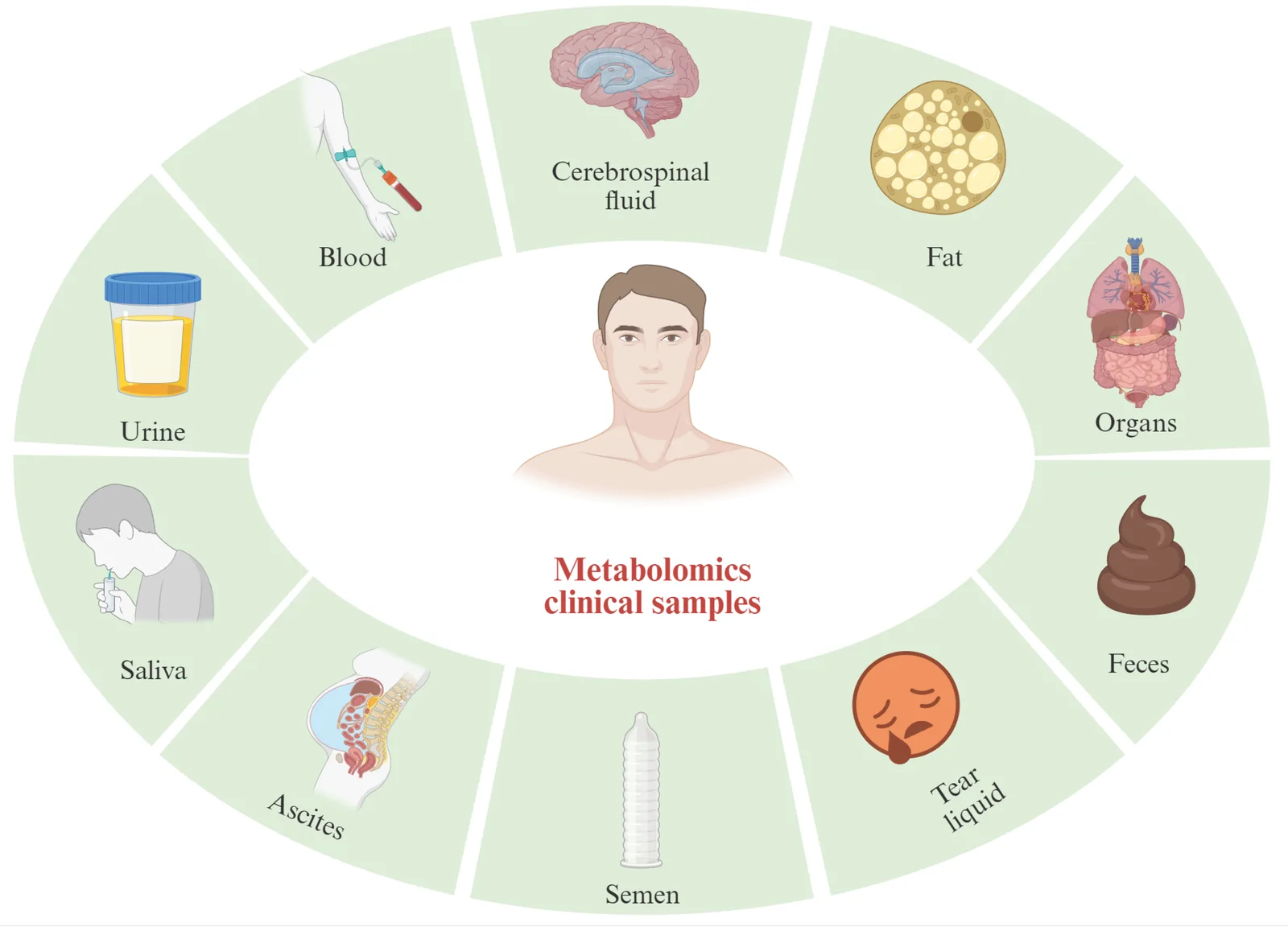
** Samples frequently utilized in clinical metabolomics investigations.** Biological matrices used in clinical metabolomics studies for the prediction and early diagnosis of T2DM, including blood, urine, saliva, feces, cerebrospinal fluid, and other sample types. Created with BioRender.com (Agreement number: IR29M9Q1LG).

**Figure 6:**
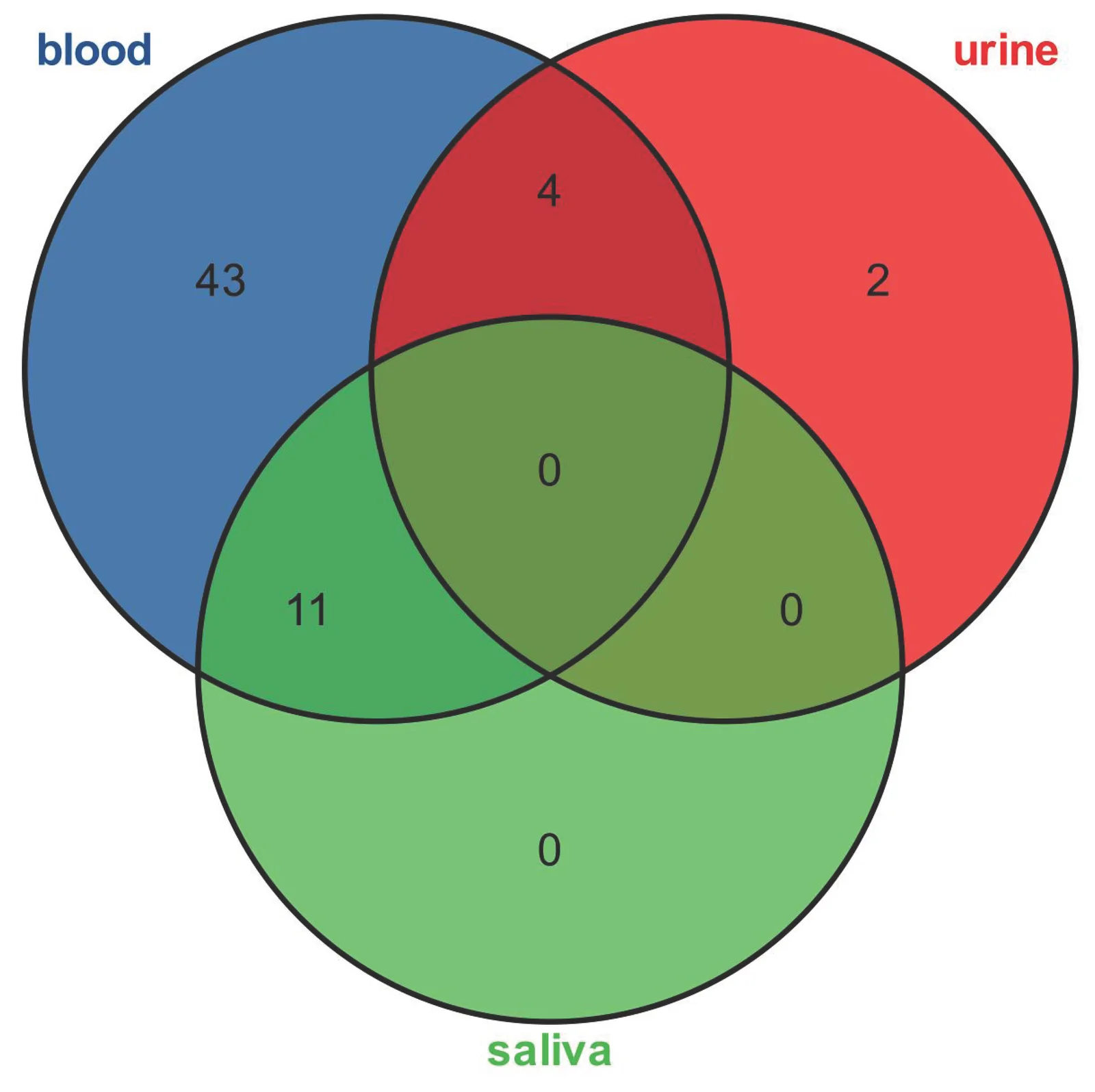
** Venn diagram illustrating the overlap of metabolites associated with the early prediction and diagnosis of T2DM.** The numbers within each section represent the count of unique or shared metabolites identified across the 66 reviewed studies. In this figure, “blood” refers to the combined category of serum and plasma. Due to the limited number of reported overlapping metabolites in fecal and CSF studies, Figure 6 focuses on the three sample types with sufficient comparative data: blood, urine, and saliva. Blue, red, and green circles correspond to blood, urine, and saliva samples, respectively.

**Figure 7:**
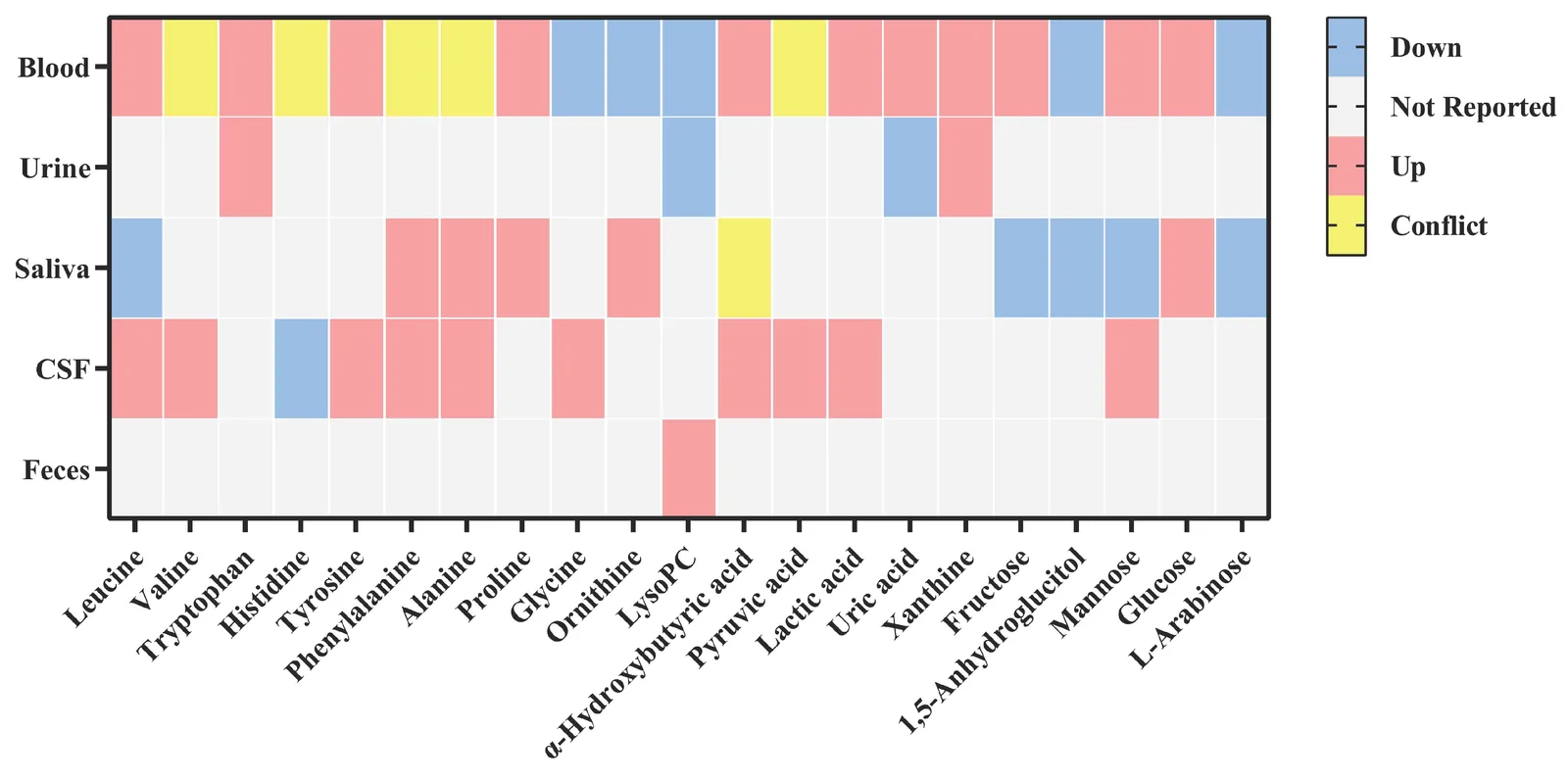
** Heatmap of abundance trends for core metabolites across multiple biological samples.** The heatmap displays representative metabolites reported in at least two distinct sample types among the 66 included studies. The colors indicate the direction of metabolite level changes in T2DM/Prediabetes groups compared to controls: Red represents upregulation; Blue represents downregulation; Yellow indicates conflicting results across different studies (both up- and downregulation reported); and White denotes that the metabolite was not reported or detected in that specific sample type.

**Table 1:** Diagnostic criteria for T2DM and prediabetes

	Authoritative institution	FPG (mmol/L)	2hPG (mmol/L)	HbA1c (%)	Reference
T2DM	WHO	≥ 7.0	≥ 11.1	≥ 6.5	43
	ADA	≥ 7.0	≥ 11.1	≥ 6.5	44
	IDF	≥ 7.0	≥ 11.1	≥ 6.5	45
Prediabetes	WHO	6.1-6.9	7.8-11.0	NR	43
	ADA	5.6-6.9	7.8-11.0	5.7-6.4	44
	IDF	5.6/6.1-6.9	7.8-11.0	5.7-6.4/NR	45

NR: Data not reported in the original publication; WHO: World Health Organization; ADA: American Diabetes Association; IDF: International Diabetes Federation; FPG: fasting plasma glucose; 2hPG: 2-h plasma glucose; HbA1c: glycated hemoglobin

**Table 2:** Summary of characteristics of the 66 included studies

Characteristic	Category	Number of Studies (n)	Percentage (%)
Study Design	Predictive (Prospective)	33	50.0
	Diagnostic (Cross-sectional)	33	50.0
Sample Type	Blood (Plasma/Serum)	54	81.8
	Urine	1	1.5
	Saliva	2	3.0
	Feces	2	3.0
	CSF	1	1.5
	Multi-sample	6	9.1
Analytical Platform	LC-MS based	36	54.5
	NMR	13	19.7
	GC-MS based	3	4.5
	Other	8	12.1
	Combined	6	9.1
Validation Strategy	Internal Validation / No Validation	49	74.2
	Independent External Validation	17	25.8
Sample Size	< 100 participants	15	22.7
	100-1000 participants	30	45.5
	> 1000 participants	21	31.8
Geographic Region	Europe	26	39.4
	Asia & Middle East	27	40.9
	North America	9	13.6
	Multi-continental	4	6.1

Abbreviations: CSF, cerebrospinal fluid; GC-MS, gas chromatography-mass spectrometry; LC-MS, liquid chromatography-mass spectrometry; NMR, nuclear magnetic resonance.

**Table 3:** Reported metabolite markers for the prediction and early diagnosis of T2DM

Metabolic pathway	Metabolite	Change	Sample	Collection time	Metabolomics technology	Study design	Reference
Amino acid	Leucine	↑	plasma	not eating or drinking since 8:00 the night before (≥10h)	*LC-MS*	*Cross-sectional*	48
		↑	serum	after a 10-hour overnight fast; fasting was not required before blood collection	**GC-MS; LC-MS**	*Cross-sectional/* Prospective	90,57
		↑	CSF	fasting	NMR	Cross-sectional	101
		↓	saliva	in the afternoon	UHPLC-MS/MS; GC-MS/MS	Cross-sectional	98
	Valine	↑	plasma	not eating or drinking since 8:00 the night before (≥10h); after a 12-h fasting period; fasting; fast for 8-12 h	*LC-MS*; UPLC-MS; LC-MS/MS; MS	Cross-sectional	48,67,54,69
		↑	serum	after a 10-hour overnight fast, fasting was not required before blood collection	**GC-MS; LC-MS**	Cross-sectional/ Prospective	90,57
		↓	serum	after 12 h fasting, in the morning	**NMR**	Cross-sectional	103
		↑	CSF	fasting	NMR	Cross-sectional	101
	Tryptophan	↑	blood	in the morning, between 8:00 and 10:30 am, after not eating for at least 10 hours	UPLC	Cross-sectional	84
		↑	plasma	fasting	LC-MS/MS	Cross-sectional	54
		↑	urine	after an overnight fast under standardized conditions	UPLC-QTOF-MS	Cross-sectional	12
	Histidine	↑	plasma	after an overnight fast, not eating or drinking since 8:00 the night before (≥10h)	MS	Cross-sectional	48
		↓	CSF	fasting	NMR	Cross-sectional	101
		↓	plasma	overnight fast	UPLC-MS/MS	Prospective	77
		↓	serum	after 12 h fasting, in the morning	NMR	Cross-sectional	103
	Tyrosine	↑	plasma	Fasting; Fasting; mostly non-fasting	LC-MS; LC-MS/MS; NMR	Prospective/Cross-sectional/Prospective	64,54,102
		↑	serum	mostly non-fasting	NMR	Prospective	102
		↑	CSF	fasting	NMR	Cross-sectional	101
	Phenylalanine	↑	serum	fasting was not required before blood collection; fasting; after a 10-hour overnight fast	LC-MS**; LC-MS; GC-MS**	Prospective/Prospective/Cross-sectional	57,89,90
		↑	plasma	fasting after a 12-h fasting period	LC-MS; UPLC-MS; LC-MS/MS	Prospective/Cross-sectional/Cross-sectional	64,67,54
		↑	CSF	fasting	NMR	Cross-sectional	101
		↓	serum	overnight fasting	NMR	Cross-sectional	88
		↑	saliva	in the afternoon	UHPLC-MS/MS; GC-MS/MS	Cross-sectional	98
	Alanine	↑	serum	after a 10-hour overnight fast.	GC-MS	Cross-sectional	90
		↓	serum	after 12 h fasting, in the morning	NMR	Cross-sectional	103
		↑	plasma	Fasting; fasting (≥8 h)	LC-MS; NMR	Cross-sectional/Prospective/Prospective/Cross-sectional	54,63,64,101
		↑	CSF	fasting	NMR	Cross-sectional	101
		↓	plasma	overnight fast of at least 8 h	FIA-MS/MS; LC-MS/MS	Cross-sectional	68
		↑	saliva	in the afternoon	UHPLC-MS/MS; GC-MS/MS	Cross-sectional	98
	Proline	↑	serum	after a 10-hour overnight fast.	GC-MS; LC-MS	Cross-sectional	90
		↑	plasma	after a 12-h fasting period; fasting; fast for 8-12 h	UPLC-MS; LC-MS/MS; MS	Cross-sectional	67,54,69
		↑	saliva	in the afternoon	UHPLC-MS/MS; GC-MS/MS	Cross-sectional	98
	Glutamic acid	↑	serum	after a 10-hour overnight fast; fasting; after 12 h fasting, in the morning	GC-MS; LC-MS; NMR	Cross-sectional/Prospective/Cross-sectional	90,89,103
		↓	serum	overnight fasting	NMR	Cross-sectional	88
		↑	plasma	fasting	LC-MS/MS	Cross-sectional	54
	Glycine	↓	serum	fasting	LC-MS	Prospective	79,89
		↑	CSF	fasting	NMR	Cross-sectional	101
		↓	plasma	fasting; fast for 8-12 h	LC-MS/MS; MS	Cross-sectional	54,69
	Lysine	↑	plasma	after a 12-hour fasting period	UPLC-QTOF-MS	Cross-sectional	70
		↓	serum	overnight fasting	NMR	Cross-sectional	88
	Methionine	↓	serum	overnight fasting	NMR	Cross-sectional	88
		↑	plasma	fasting	LC-MS/MS	Cross-sectional	54
	Serine	↓	blood	in the morning, between 8:00 and 10:30 am, after not eating for at least 10 hours	UPLC	Cross-sectional	84
		↓	serum	fasting serum	*LC-MS*	Prospective	89
		↓	plasma	fasting	LC-MS/MS	Cross-sectional	54
		↓	plasma	fast for 8-12 h	MS	Cross-sectional	69
	Arginine	↑	serum	fasting	*LC-MS*	Prospective	89
		↓	plasma	Fasting; fasted overnight for at least 12 h	LC-MS/MS	Cross-sectional	54,11
	Ornithine	↓	plasma	fasting	LC-MS/MS	Cross-sectional	54
		↑	saliva	in the afternoon	UHPLC-MS/MS; GC-MS/MS	Cross-sectional	98
	Dimethylglycine	↑	plasma	fasting	*LC-MS*	Prospective	62
	**2-Pyrrolidone glycine**	↑	plasma	following a 12-hour overnight fast, from 6:30 to 9:30	UPLC-QTOF-MS	Prospective	73
	Cystine	↑	plasma	no food or drink since 20:00 the night before (≥10h)	*LC-MS*	Cross-sectional	48
	Homocysteine	↓	blood	in the morning, between 8:00 and 10:30 am, after not eating for at least 10 hours	UPLC	Cross-sectional	84
	Aspartic acid	↑	plasma	overnight fast	UPLC-MS/MS	Prospective	77
	Threonine	↓	plasma	fasting	LC-MS/MS	Cross-sectional	54
		↓	serum	after 12 h fasting, in the morning	NMR	Cross-sectional	103
	Beta-threonine	↓	blood	in the morning (8:00-10:30 am) after a period of ≥10 hrs fasting	UPLC	Cross-sectional	84
Lipid	Palmitic acid	↑	serum	after a 10-hour overnight fast	GC-MS; LC-MS	Cross-sectional	90
		↑	serum	fasting	UPLC-MS	Cross-sectional	92
	Stearic acid	↑	plasma	following a standardized overnight fast	UPLC-QTOF-MS	Cross-sectional	12
		↑	serum	after a 10-hour overnight fast	GC-MS; LC-MS	Cross-sectional	90
	Acetyl carnitine	↑	plasma	not eating or drinking since 8:00 the night before (≥10h); fasting	*LC-MS*; LC-MS/MS	Cross-sectional	48,54
		↑	serum	fasting	LC-MS	Prospective	85
	Lysophosphatidylcholine (LysoPC)	↓	plasma	following a standardized overnight fast	UPLC-QTOF-MS	Cross-sectional	12
		↓	urine	following a standardized overnight fast	UPLC-QTOF-MS	Cross-sectional	12
		↑	fecal	fasting	LC-MS; GC-MS	Cross-sectional	100
	Sphingomyelin	↓	serum	after a 10-hour overnight fast	GC-MS; LC-MS	Cross-sectional	90
		↑	serum	in the morning	UPLC-MS	Cross-sectional	92
		↓	plasma	overnight fast of at least 8 h	FIA-MS/MS; LC-MS/MS	Cross-sectional	68
	Deoxycholic acid	↑	plasma, serum	NR	LC-MS/MS	Prospective	13
		↑	serum	in the morning	UPLC-MS	Cross-sectional	92
	Chenodeoxycholic acid	↑	plasma	following a standardized overnight fast	UPLC-QTOF-MS	Cross-sectional	12
		↑	serum	in the morning	UPLC-MS	Cross-sectional	92
	Myristic acid	↑	serum	after a 10-hour overnight fast	GC-MS; LC-MS	Cross-sectional	90
	Oleic acid ester	↑	plasma	following a standardized overnight fast	UPLC-QTOF-MS	Cross-sectional	12
	Docosahexaenoic acid	↓	plasma	NR	UHPLC-MS/MS; GC-MS/MS	Prospective	66
	3-Hydroxybutyrylcarnitine	↑	plasma	fasting	LC-MS/MS	Cross-sectional	54
	LysoPC(14:0)	↑	plasma	after a 12-h fasting period	UPLC-QTOF-MS	Cross-sectional	70
	Decanoylcarnitine	↑	plasma	after a 12-h fasting period	UPLC-QTOF-MS	Cross-sectional	70
	Glycocholic acid	↓	fecal	fasting	LC-MS; GC-MS	Cross-sectional	100
	VLDL	↑	plasma	NR	NMR	Prospective	65
	HDL cholesterol	↓	plasma	NR	NMR	Prospective	65
	Cholesterol sulfate	↑	serum	in the morning	UPLC-MS	Cross-sectional	92
	Diacylglycerol	↓	plasma	Overnight fasting	LC-MS	Prospective	79
Organic acid	α-hydroxybutyric acid	↑	plasma	not eating or drinking since 8:00 the night before (≥10h)	*LC-MS*; LC-MS	Cross-sectional /Prospective	48,62
		↑	CSF	not eating or drinking since 8:00 the night before (≥10h)	NMR	Cross-sectional	101
		↓	saliva	in the afternoon	UHPLC-MS/MS; GC-MS/MS	Cross-sectional	98
		↑	serum	fasting	UHPLC-MS; GC-MS; UHPLC-MS/MS	Prospective	86
		↑	saliva	fasting	LC-MS; GC-MS	Cross-sectional	97
	3-Hydroxybutyric acid	↑	serum	after a 10-hour overnight fast; mostly non-fasting	GC-MS; LC-MS; NMR	Cross-sectional/ Prospective	90,102
		↑	plasma	overnight fast of at least 8 h; mostly non-fasting	FIA-MS/MS; LC-MS/MS; NMR	Cross-sectional/ Prospective	68,102
	Pyruvic acid	↓	serum	after a 10-hour overnight fast	GC-MS; LC-MS	Cross-sectional	90
		↑	serum	mostly non-fasting	NMR	Prospective	102
		↑	CSF	Fasting	NMR	Cross-sectional	101
		↑	plasma	fasting (≥8 h); mostly non-fasting	NR; NMR	Prospective/ Prospective	63,102
	Acetic acid	↑	plasma	Fasting	NMR	Cross-sectional	101
		↓	plasma	mostly non-fasting	NMR	Prospective	102
		↓	serum	mostly non-fasting	NMR	Prospective	102
	Citric acid	↓	plasma	Fasting	NMR	Cross-sectional	101
		↑	plasma	mostly non-fasting	NMR	Prospective	102
		↑	serum	mostly non-fasting	NMR	Prospective	102
	Lactic acid	↑	plasma	mostly non-fasting	NMR	Prospective	102
		↑	serum	mostly non-fasting	NMR	Prospective	102
		↑	CSF	Fasting	NMR	Cross-sectional	101
	Trimethyluric acid	↑	plasma	overnight fast of at least 8 h	FIA-MS/MS; LC-MS/MS	Cross-sectional	68
	Ethyl lactate	↑	serum	at 7:00-8:30 AM after an overnight fast	UHPLC/Q-Orbitrap-HRMS	Cross-sectional	58
	2-Hydroxy-2,4-pentadienoic acid	↑	serum	at 7:00-8:30 AM after an overnight fast	UHPLC/Q-Orbitrap-HRMS	Cross-sectional	58
	Isocitric acid	↑	plasma	fasting (≥8 h)	NR	Prospective	63
	3-Methyl-2-oxobutyrate	↑	serum	after 12 hours of fasting	NR	Cross-sectional	87
Nucleotide	Uric acid	↑	plasma	fasting	LC-MS	Prospective	62
		↓	urine	following a standardized overnight fast	UPLC-QTOF-MS	Cross-sectional	12
	Xanthine	↑	plasma	fasting	LC-MS	Prospective	62
		↑	urine	following a standardized overnight fast	UPLC-QTOF-MS	Cross-sectional	12
	Methyluric acid	↓	urine	following a standardized overnight fast	UPLC-QTOF-MS	Cross-sectional	12
	Methylxanthine	↓	urine	following a standardized overnight fast	UPLC-QTOF-MS	Cross-sectional	12
Carbohydrates	Fructose	↑	serum	after a 10-hour overnight fast	GC-MS; LC-MS	Cross-sectional	90
		↓	saliva	in the afternoon	UHPLC-MS/MS; GC-MS/MS	Cross-sectional	98
	1,5-Anhydroglucitol	↓	plasma	NR	**UHPLC-MS/MS**; **GC-MS/MS**	Prospective	66
		↓	saliva	fasting; in the afternoon	GC-MS; LC-MS; UHPLC-MS/MS; GC-MS/MS	Cross-sectional	97,98
	Mannose	↑	plasma	NR	**UHPLC-MS/MS; GC-MS/MS**	Prospective	66
		↓	saliva	in the afternoon	UHPLC-MS/MS; GC-MS/MS	Cross-sectional	98
		↑	serum	Fasting; after 12 h fasting, in the morning	UHPLC-MS; GC-MS; UHPLC-MS/MS; NMR	Prospective/ Cross-sectional	86,103
		↑	CSF	fasting	NMR	Cross-sectional	101
	Glucose	↑	serum	Fasting; after 12 h fasting, in the morning; mostly non-fasting	UHPLC-MS; GC-MS; UHPLC-MS/MS; NMR	Prospective /Cross-sectional/Prospective	86,103,102
		↑	plasma	mostly non-fasting	NMR	Prospective	102
		↑	saliva	fasting	LC-MS; GC-MS	Cross-sectional	97
		↑	saliva	in the afternoon	UHPLC-MS/MS; GC-MS/MS	Cross-sectional	98
	L-Arabinose	↓	serum	at 7:00-8:30 AM after an overnight fast	UHPLC/Q-Orbitrap-HRMS	Cross-sectional	58
		↓	saliva	in the afternoon	UHPLC-MS/MS; GC-MS/MS	Cross-sectional	98
	N-acetylglycoprotein	↑	plasma	Fasting; non-fasting	NMR; NMR	Cross-sectional/ Prospective	101,104

NR: Data not reported in the original publication; MS: Mass Spectrometry; FIA-MS/MS: Flow Injection Analysis-Tandem Mass Spectrometry; GC-MS: Gas Chromatography-Mass Spectrometry; GC-MS/MS: Gas Chromatography-Tandem Mass Spectrometry; LC-MS: Liquid Chromatography-Mass Spectrometry; LC-MS/MS: Liquid Chromatography-Tandem Mass Spectrometry; NMR: Nuclear Magnetic Resonance Spectroscopy; UPLC: Ultra Performance Liquid Chromatography; UPLC-MS: Ultra Performance Liquid Chromatography-Mass Spectrometry; UPLC-QTOF-MS: Ultra Performance Liquid Chromatography-Quadrupole Time-of-Flight Mass Spectrometry; UHPLC-MS: Ultra High Performance Liquid Chromatography-Mass Spectrometry; UHPLC-MS/MS: Ultra High Performance Liquid Chromatography-Tandem Mass Spectrometry; UHPLC/Q-Orbitrap-HRMS: Ultra High Performance Liquid Chromatography/Quadrupole-Orbitrap High Resolution Mass Spectrometry. Note: Serum and plasma are presented separately in Table 3, but were grouped together as “blood” in Figure 6.
