# Supplementary material for: Metabolomics in the Prediction and Early Diagnosis of Type 2 Diabetes Mellitus: Advances and Applications
Source: Int J Med Sci. 2026 Sep 11;23(11):3336–55. doi: 10.7150/ijms.126351 (PMC13685039; doi:10.7150/ijms.126351)
Supplement: Supplementary file 1 [file ijmsv23p3336s1.pdf]

Supplementary Table S1

Research on the prediction and early diagnosis of T2DM based on metabolomics

Sample	Region/Ethnicity	Number of people	Gender	Age (years)	Analytical Platform	Diagnostic criteria	Study design	Reference
Plasma	Europe	3841	Male, Female	30–60	UHPLC-MS/MS	Based on the diagnostic criteria of the WHO	Prospective	[49]
Plasma	Finland	11896	Male, Female	24–45	NMR	NR	Prospective	[61]
Plasma	Finland	5181	Male	45–73	LC-MS	NR	Prospective	[62]
Plasma	Spain	889	Male, Female	55–80	NMR	Based on the diagnostic criteria of the ADA.	Prospective	[63]
Plasma	African-American	55	Female	NR	LC-MS	NR	Cross-sectional	[48]
Plasma	American Indians	431	Male, Female	≥14	LC-MS	Based on the diagnostic criteria of the ADA, 2003.	Prospective	[51]
Plasma	China (Hubei Province, Jiangsu Province)	1559	Male, Female	NR	LC-MS	Based on the diagnostic criteria of the ADA or WHO, or patients had previously used diabetes medications.	Prospective	[64]
Plasma	China (Liaoning Province)	210	Male, Female	19–77	MS/MS	Based on the diagnostic criteria of the ADA, 2018.	Cross-sectional	[8]
Plasma	China (multicenter nationwide)	1671	Male, Female	30–79	NMR	NR	Prospective	[65]
Plasma	China (Yinchuan City)	80	Male, Female	NR	UPLC-MS/MS	Based on the diagnostic criteria of the ADA.	Cross-sectional	[11]
Plasma	China	1105	Male, Female	40–74	UHPLC-MS/MS; GC-MS/MS	Based on the diagnostic criteria of the ADA or WHO, or patients had previously used diabetes medications.	Prospective	[66]
Plasma	China (Suzhou City)	293	Male, Female	NR	LC-MS	Based on the diagnostic criteria of the WHO.	Cross-sectional	[56]

Sample	Region/Ethnicity	Number of people	Gender	Age (years)	Analytical Platform	Diagnostic criteria	Study design	Reference
Plasma	South Korea	130	Male, Female	30–70	UPLC-MS	Based on the diagnostic criteria of the ADA	Cross-sectional	[67]
Plasma	United Arab Emirates	100	Male, Female	18–60	FIA-MS/MS; LC-MS/MS	NR	Cross-sectional	[68]
Plasma	Iran	1102	Male, Female	Adults	MS	NR	Cross-sectional	[69]
Plasma	South Korea	53	Male	35–65	UPLC-QTOF-MS	Based on the diagnostic criteria of the ADA or WHO.	Cross-sectional	[70]
Plasma	United States	1622	Male, Female	NR	LC-MS	Based on the diagnostic criteria of the ADA or WHO, or if the individual is currently undergoing medical treatment for diabetes.	Prospective	[7]
Plasma	Europe	1623	Male, Female	30–60	LC-MS/MS	Based on the diagnostic criteria of the ADA.	Cross-sectional	[71]
Plasma	Germany	640	Male, Female	NR	MS	Based on the diagnostic criteria of the ADA.	Prospective	[72]
Plasma	Sweden (Gothenburg)	629	Female	64	GC-MS/MS	Based on the diagnostic criteria of the WHO.	Prospective	[59]
Plasma	China	108	Male, Female	18–76	UPLC-QTOF-MS	NR	Prospective	[73]
Plasma	United States	1150	Male, Female	40–65	LC-MS/MS	Based on the diagnostic criteria of the ADA or WHO, or patients had previously used diabetes medications, including insulin.	Prospective	[74]
Plasma	United States (Cleveland)	90	Male, Female	20–59	HPLC-UV; LC-MS/MS	NR	Cross-sectional	[75]
Plasma	Netherlands (Leiden region)	533	Male, Female	45–65	FIA-ESI-MS/MS	Based on the diagnostic criteria of the WHO, and with no history of IFG or T2DM.	Cross-sectional	[76]
Plasma	United States	140	Male, Female	18–65	UPLC-MS/MS	Based on the diagnostic criteria of the ADA.	Prospective	[77]
Plasma	United States	106	Male, Female	25–75	LC-MS/MS	Based on the diagnostic criteria of the ADA.	Prospective	[50]
Plasma	Spain	700	Male, Female	66.5 (SD:5.8)	LC-MS/MS	Based on the diagnostic criteria of the ADA.	Prospective	[78]

Sample	Region/Ethnicity	Number of people	Gender	Age (years)	Analytical Platform	Diagnostic criteria	Study design	Reference
Plasma	Germany (Pomerania)	827	Male, Female	NR	MS	Based on the diagnostic criteria of the WHO.	Cross-sectional	[55]
Plasma	Sweden	374	Male, Female	46 ± 6	LC-MS	Based on the diagnostic criteria of the WHO	Prospective	[79]
Plasma	United Kingdom	488	Female	30–50	LC-MS	Based on the diagnostic criteria of the ADA.	Prospective	[46]
Plasma	United Kingdom	65684	Male, Female	40–69	NMR	NR	Prospective	[60]
Plasma	Iran	412	Male, Female	45–90	LC-MS/MS	NR	Cross-sectional	[54]
Plasma	Spain	199	Male, Female	8–23	LC-MS	Based on the diagnostic criteria of the ADA.	Prospective	[14]
Plasma	India	445	Male, Female	NR	LC-MS/MS	Based on the diagnostic criteria of the ADA	Cross-sectional	[80]
Plasma	Netherlands	2776	Male, Female	NR	LC-MS; NMR	Based on the diagnostic criteria of the ADA or WHO, or if the individual is currently undergoing medical treatment for diabetes.	Prospective	[81]
Plasma/ Urine	United Kingdom	2204	Female	NR	MS	Based on the diagnostic criteria of the ADA.	Cross-sectional	[82]
Plasma/ Urine	China/Germany	51	NR	46.9 (SE:11.9)	UPLC-QTOF-MS	Based on the diagnostic criteria of the WHO.	Cross-sectional	[12]
Plasma/ Serum	Sweden/Germany	4593	Male, Female	Twin Gene:68.0 ± 8.1<br>ULSAM:71.0 ± 0.6<br>PIVUS:70.2 ± 0.2<br>KORA S4:63.1 ± 0.4	UPLC-QTOF-MS	Based on the diagnostic criteria of the ADA.	Prospective	[13]
Plasma/ Serum	France	1172	Male, Female	NR	GC-MS; LC-MS/MS	Based on the diagnostic criteria of the ADA or WHO, and patients had previously used diabetes medications.	Prospective	[83]
Blood	Saudi Arabia	67	Male, Female	24–80	UPLC	NR	Cross-sectional	[84]
Serum	Germany(Augsburg)	4297	Male, Female	NR	LC-MS	NR	Prospective	[85]

Sample	Region/Ethnicity	Number of people	Gender	Age (years)	Analytical Platform	Diagnostic criteria	Study design	Reference
Serum	Finland	543	Male, Female	NR	UHPLC-MS; GC-MS; UHPLC-MS/MS	NR	Prospective	[86]
Serum	Sweden	53	Male	42 (SD:8)	MRS	NR	Cross-sectional	[87]
Serum	Italy	33	Male, Female	55–72	NMR	NR	Cross-sectional	[88]
Serum	China (Beijing City)	149	Male, Female	NGT:48.94 ± 10.59; IFG:49.54 ± 9.58; T2DM:50.31 ± 9.94	UHPLC-MS	Based on the diagnostic criteria of the WHO.	Cross-sectional	[58]
Serum	China	394	Male, Female	45–74	LC-MS	NR	Prospective	[57]
Serum	Singapore (participants were of Chinese, Malay, and Indian ethnicity)	3313	Male, Female	48 (SD:11.9)	LC-MS	NR	Prospective	[89]
Serum	Singapore (Chinese, Malay, Indian)	111	Male, Female	30–63	GC-MS	Based on the diagnostic criteria of the ADA or WHO.	Cross-sectional	[90]
Serum	Poland	932	Male, Female	NR	CE-MS	Based on the diagnostic criteria of the ADA.	Cross-sectional	[91]
Serum	Spain	64	Male, Female	Adults	LC-MS/MS; FIA-MS/MS	Based on the diagnostic criteria of the ADA.	Cross-sectional	[47]
Serum	China (Heilongjiang Province)	134	NR	35–80	UPLC-MS	Based on the diagnostic criteria of the ADA, 2015.	Cross-sectional	[92]
Serum	Finland	200	Male, Female	40–64	UHPLC-QTOF-MS	Based on the diagnostic criteria of the WHO, 1985.	Prospective	[93]

Sample	Region/Ethnicity	Number of people	Gender	Age (years)	Analytical Platform	Diagnostic criteria	Study design	Reference
Serum	Saudi Arabia	72	Male, Female	Lean:25.7 ± 5.77; Obese:36.2 ± 12.12; T2DM:49.4 ± 12.12	LC-MS	NR	Cross-sectional	[94]
Serum	China (multicenter nationwide)	1705	Male, Female	NR	UHPLC-MS	Based on the diagnostic criteria of the WHO.	Cross-sectional	[95]
Serum	China	481	Male, Female	NR	UHPLC-MS/MS	Based on the diagnostic criteria of the WHO,1999.	Cross-sectional	[53]
Serum /Feces	British/European Americans	1018	Female/Male, Female	TwinsUK: 65.19 ± 7.74; ARIC: 54.56 ± 5.74	UHPLC-MS/MS	Based on the diagnostic criteria of the ADA or WHO, or individuals with a physician's letter confirming diagnosis or use of diabetes medication.	Prospective	[96]
Urine	Dutch ancestry	69	Male	30–70	GC-MS	Based on the diagnostic criteria of the WHO.	Prospective	[52]
Saliva	New York	161	Male, Female	18–65	LC-MS; GC-MS	NR	Cross-sectional	[97]
Saliva	Qatar	369	Male, Female	T2DM:53.8 (35.0–70.7); Controls:38.5 (23.6–62.3)	UHPLC-MS/MS; GC-MS/MS	NR	Cross-sectional	[98]
Feces	China (Xinjiang)	60	NR	30–60	LC-MS	Based on the diagnostic criteria of the ADA, 2015.	Cross-sectional	[99]
Feces	China (Shandong Province)	100	Male, Female	NR	LC-MS; GC-MS	Based on the diagnostic criteria of the ADA or WHO.	Cross-sectional	[100]
CSF	China (Taiwan Province)	76	Male, Female	20–70	NMR	NR	Cross-sectional	[101]

Sample	Region/Ethnicity	Number of people	Gender	Age (years)	Analytical Platform	Diagnostic criteria	Study design	Referen ce
Plasma /Serum	United Kingdom/ Germany	86232	Male, Female	37–73	NMR	NR	Prospective	[102]
Serum	India	77	Male, Female	38–64	NMR	Based on the diagnostic criteria of the ADA or WHO.	Cross-sectional	[103]
Plasma	United Kingdom	98831	Male, Female	40–69	NMR	Incident T2DM identified via hospital records and death registers using ICD-9 and ICD-10 codes over a 12-year follow-up.	Prospective	[104]
Plasma	India	94	Male, Female	18–40	LC-MS/MS	Based on the diagnostic criteria of the ADA, 2019	Prospective	[105]

NR: Data not reported in the original publication; CSF: cerebrospinal fluid
